# Impact of A-Site Cation Deficiency on Charge Transport in La_0.5−*x*_Sr_0.5_FeO_3−_*_δ_*

**DOI:** 10.3390/ma14205990

**Published:** 2021-10-12

**Authors:** Oleg V. Merkulov, Ruslan R. Samigullin, Alexey A. Markov, Mikhail V. Patrakeev

**Affiliations:** 1 Institute of Solid State Chemistry, UB RAS, 620990 Ekaterinburg, Russia; markov@ihim.uran.ru (A.A.M.); patrakeev@ihim.uran.ru (M.V.P.); 2 Institute of Solid State Chemistry and Mechanochemistry, SB RAS, 630128 Novosibirsk, Russia; 3 Chemistry Department, Moscow State University, 119991 Moscow, Russia; ruslan.samigullin@chemistry.msu.ru

**Keywords:** cation deficiency, strontium hexaferrite impurity, ceramics density, charge carrier mobility

## Abstract

The electrical conductivity of La_0.5−*x*_Sr_0.5_FeO_3−*δ*_, investigated as a function of the nominal cation deficiency in the A-sublattice, *x*, varying from 0 to 0.02, has demonstrated a nonlinear dependence. An increase in the *x* value from 0 to 0.01 resulted in a considerable increase in electrical conductivity, which was shown to be attributed mainly to an increase in the mobility of the charge carriers. A combined analysis of the defect equilibrium and the charge transport in La_0.5−*x*_Sr_0.5_FeO_3−*δ*_ revealed the increase in the mobility of oxygen ions, electrons, and holes by factors of ~1.5, 1.3, and 1.7, respectively. The observed effect is assumed to be conditioned by a variation in the oxide structure under the action of the cationic vacancy formation. It was found that the cation deficiency limit in La_0.5−*x*_Sr_0.5_FeO_3−*δ*_ did not exceed 0.01. A small overstep of this limit was shown to result in the formation of (Sr,La)Fe_12_O_19_ impurity, which even in undetectable amounts reduced the conductivity of the material. The presence of (Sr,La)Fe_12_O_19_ impurity was revealed by X-ray diffraction on the ceramic surface after heat treatment at 1300 °C. It is most likely that the formation of traces of the liquid phase under these conditions is responsible for the impurity migration to the ceramic surface. The introduction of a cation deficiency of 0.01 into the A-sublattice of La_0.5−*x*_Sr_0.5_FeO_3−*δ*_ can be recommended as an effective means to enhance both the oxygen ion and the electron conductivity and improve ceramic sinterability.

## 1. Introduction

Perovskite-type mixed conducting oxides La_1−*x*_Sr*_x_*FeO_3−*δ*_ have attracted a considerable attention as promising electrode materials for solid oxide fuel cells (SOFC) and solid oxide electrolysis cells (SOEC), as membranes for oxygen separation and the partial oxidation of methane, and as oxygen carrier materials for chemical looping processes [1,2,3,4,5,6]. A great interest in the potential practical use of these materials has stimulated detailed studies of their structural, thermomechanical, thermodynamic, and transport properties [7,8,9,10,11,12].

The influence of the main factors on the practically valuable characteristics is well documented and understood. The acquired knowledge about the impact of strontium content, temperature, oxygen partial pressure in the gas phase on the thermal expansion, thermodynamic stability, and ion and electron conductivity enables one to adjust the functional properties of oxides according to the operational conditions. Of course, this knowledge should ensure the reliable production of the materials with reproducible properties. Nevertheless, considerable differences in the reported characteristics of the materials with identical compositions have sometimes been detected upon comparison [11,13,14]. To some extent, the observed differences can be attributed to the difference in the experimental equipment used for obtaining the data. However, the main cause is in the neglected factors that can provide a noticeable effect on the material properties, especially on their charge transport characteristics. For instance, most publications related to the transport properties of ferrites lack any information on the morphology of the materials. Concurrently, several other studies have pointed out its importance. In particular, the oxygen flux through the membrane of La_0.5_Sr_0.5_FeO_3−*δ*_ has been shown to depend strongly on the ceramic grain size, which is due to the preferable oxygen transport along the grain boundaries [15]. Several other studies have also reported the influence of the microstructure on the oxygen ion transport in the ceramics of ferrite-based oxides, which confirms the importance of ceramic morphology control [16,17,18,19].

Another commonly disregarded factor that can affect the charge transport characteristics in perovskite-type ferrites is an uncontrolled deviation from the cation stoichiometry, which occurs due to the limited reagent purity and the accuracy in the synthesis process. The importance of this factor has been demonstrated in a number of studies with an intentional cation deficiency introduction. This approach is attracting attention as an instrument for the modification of perovskite oxide properties because it does not imply the involvement of other elements and thus restricts unexpected side effects while potentially enhancing the performance of the materials. For example, the oxygen flux density through a ceramic membrane of the cation-deficient (Ba_0.5_Sr_0.5_)_1−x_Co_0.8_Fe_0.2_O_3−δ_ (*x* = 0.03) was reported to be 1.4 times higher than through a similar membrane of cation stoichiometric (Ba_0.5_Sr_0.5_)Co_0.8_Fe_0.2_O_3−δ_ [20]. The electrode polarization measured in the symmetric cell at 800 °C for the cation-deficient La_0.54_Sr_0.40_Co_0.20_Fe_0.80_O_3−δ_ was found to be 0.02 Ω cm^2^ when compared with 0.12 Ω cm^2^ for the cation stoichiometric La_0.54_Sr_0.40_Co_0.20_Fe_0.80_O_3−δ_ [21]. The detected improvement of the functional characteristics is attributed to the integral effect of cation deficiency on several oxide properties. It is known that cation deficiency can significantly affect the structure and morphology of perovskite-type oxides [22,23]. It has also been noted that introducing A-site cation deficiency into the perovskite lattice results in the creation of additional oxygen vacancies within the oxide lattice, which is the main reason for the increased activity of the oxygen reduction reaction. In addition, the cation deficiency increases the free volume of the perovskite unit cell, which benefits oxygen mobility [24,25].

Therefore, a comprehensive understanding of the processes and the mechanisms associated with the cation deficiency impact on the oxygen ion and the electron transport in perovskite-type ferrites would be helpful for the development of novel functional materials for high-temperature electrochemical applications. Since the compounds of the La_1−*x*_Sr*_x_*FeO_3−*δ*_ series are among the most promising functional materials for high-temperature electrochemical applications, the impact of the cation deficiency on the charge transport in these oxides is of particular interest.

It has been shown that the *x* = 0.5 composition demonstrates the highest transport characteristics in the La_1−*x*_Sr*_x_*FeO_3−*δ*_ series [26,27]. Therefore, this article is focused on the study of the microstructure and the charge transport in the oxides of the La_0.5−*x*_Sr_0.5_FeO_3−*δ*_ series depending on the nominal deficiency in the A-sublattice.

## 2. Experimental

A series of perovskite-type La_0.5−_*_x_*Sr_0.5_FeO_3−*δ*_ with a nominal deficiency in the A-sublattice of *x* = 0, 0.005, 0.010, 0.015, and 0.020 was synthesized via the glycine–nitrate route from SrCO_3_ (99.9%), La_2_O_3_ (99.3%), and Fe (99.4%). Strontium carbonate and lanthanum oxide were precalcined at 600 and 950 °C, respectively, in order to remove adsorbed water. The reagents taken in the necessary proportion were dissolved in nitric acid, then glycine was added to the solution with 50% excess to nitrates, and the mixture was heated until self-ignition, which occurred after complete water evaporation. The combustion product was calcined at 900 °C; for 10 h to eliminate traces of organics and carbon, and then thoroughly milled in a mortar with alcohol. Ceramic discs were obtained by uniaxial pressing of the oxide powder at a pressure of about 200 MPa and subsequent sintering at 1300 °C for 10 h. In order to clarify the effect of sintering temperature on the ceramic density, discs of *x* = 0 and *x* = 0.005 compositions were also obtained by sintering at 1400 and 1500 °C for 10 h.

Rectangular bars of ~2 mm × 2 mm × 15 mm were cut from the ceramic discs to measure the electrical conductivity and the relative expansion at heating, whereas the rest of the ceramics were ground for use in X-ray diffraction (XRD) and thermogravimetric (TG) analyses.

The XRD patterns were recorded at room temperature on a Shimadzu diffractometer (Shimadzu, Kyoto, Japan) with CuKα-radiation. The structural parameters were calculated using GSAS-II software [28]. The surface morphology of the ceramic samples was examined by a JEOL JSM 6390LA scanning electron microscope (SEM, JEOL Ltd., Tokyo, Japan) equipped with a JEIOL JED 2300 energy-dispersion spectrometer (EDS). Before use in the SEM analysis, the ceramic specimens were polished, and some were subjected to heat treatment at 1300 °C for 2 h to obtain a clearly distinguishable grain relief.

The oxygen content variations in La_0.5–*x*_Sr_0.5_FeO_3–*δ*_ with temperature were studied in air with a Setaram TG-92 thermal analyzer (Setaram, Lyon, France) in the cooling mode at a rate of 1 °C/min after equilibration of the sample with the ambient atmosphere at 950 °C for 5 h. Thermal expansion measurements in air were carried out with a Linseis L75 dilatometer (Linseis Messgeräte GmbH, Selb, Germany) at a heating rate of 5 °C/min.

The electrical conductivity of La_0.5–*x*_Sr_0.5_FeO_3–*δ*_ was measured by the four-probe dc method on a ceramic sample placed inside an electrochemical cell of yttria-stabilized zirconia (YZS) with an oxygen pump and a sensor, which allowed changing and measuring oxygen partial pressure over the sample. The measurement of electrical conductivity was carried out in the air atmosphere in cooling from 950 °C at a rate of 3 °C/min. In addition, the conductivity was measured versus partial pressure of oxygen varying between 10^−20^ and 0.5 atm at temperatures of 750–950 °C. The measurements were carried out under isothermal conditions by a stepwise decrease in partial oxygen pressure ($p_{O_{2}}$). Experimental data were recorded only after reaching the equilibrium between the sample and the gas phase. A change in the logarithm of conductivity less than 0.01% per minute was taken as the equilibrium criterion, which provided good reproducibility of the measurement results. The $p_{O_{2}}$ interval between ~10^−10^ and 10^−4^ atm was excluded from measurements at temperatures below 950 °C because of the extremely low equilibration kinetics [27].

The oxygen content in the oxides as a function of the partial pressure of oxygen in the gas phase at different temperatures was measured by solid state coulometric titration in a double electrochemical cell. Details of the experiment can be found elsewhere [29,30].

## 3. Results and Discussion

### 3.1. Material Characterization

**Figure 1:**
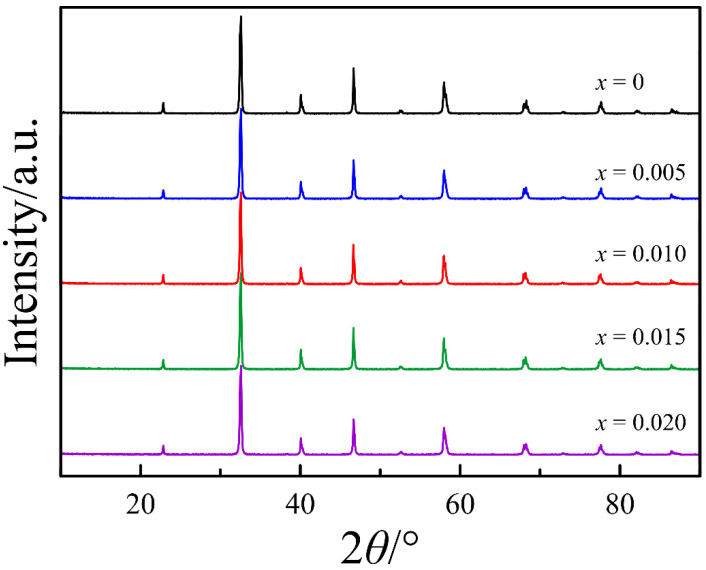
Room temperature X-ray powder diffraction patterns of La_0.5−*x*_Sr_0.5_FeO_3−*δ*_.

**Figure 2:**
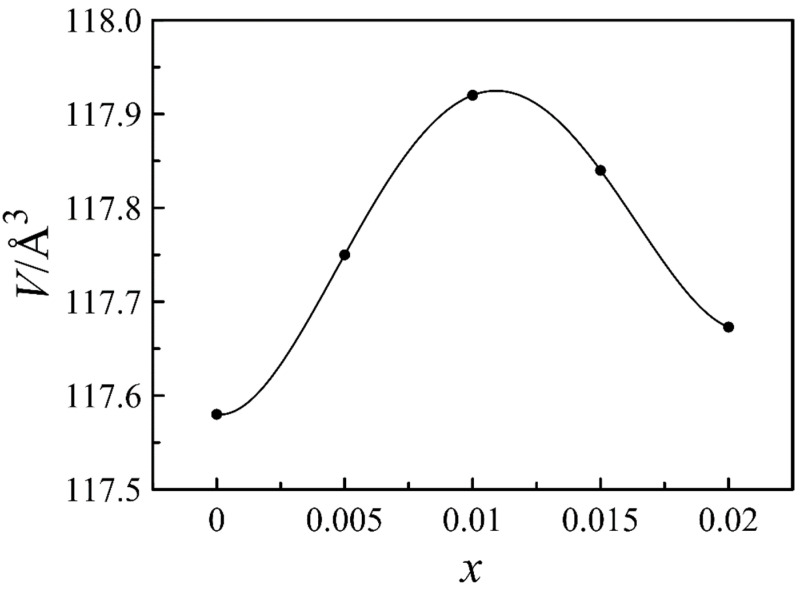
Unit cell volume of La_0.5−*x*_Sr_0.5_FeO_3−*δ*_ as a function of A-site deficiency.

The XRD patterns of La_0.5−_*_x_*Sr_0.5_FeO_3−*δ*_ (*x* = 0, 0.005, 0.010, 0.015, 0.020) in Figure 1 show the formation of single-phase oxides of the perovskite-type structure with rhombohedral symmetry (S.G. $R\bar{3}C$). The structural parameters collected in Table 1 demonstrate an extreme dependence on the nominal cation deficiency, another example of which is the unit cell volume found in Figure 2. The observed phenomenon can be explained as follows. An initial increase in the *x* value from 0 to 0.01 probably results in the formation of the cation vacancies in the A-sublattice. According to electroneutrality requirement, a decrease in the proportion of the cations should be compensated by a decrease in the oxygen content. Thus, a detected increase in the unit cell volume is an unavoidable result of a decrease in both positive and negative ions in the crystalline lattice. It should be noted that an increase in the *x* value up to 0.01 is also accompanied by the rhombohedral angle approach to 60 degrees (Table 1) which indicates a reduction of rhombohedral distortions and an improvement of the lattice symmetry. An increase in the nominal cation deficiency above 0.01 leads to a gradual return of the unit cell parameters to the initial values. According to the work of Striker et al. [22], this effect can be associated with the formation of the second phase of SrFe_12_O_19_ in concentrations below the resolution of the X-ray diffraction. The separation of the impurity phase with a high iron content results in the progressive disappearance of the cation vacancies in the A-sublattice of the perovskite phase; therefore, the structural parameters approach the values of *x* = 0 composition. Thus, it can be assumed that the real cation deficiency in the A-sublattice of La_0.5−_*_x_*Sr_0.5_FeO_3−*δ*_ cannot exceed 0.01.

**Figure 3:**
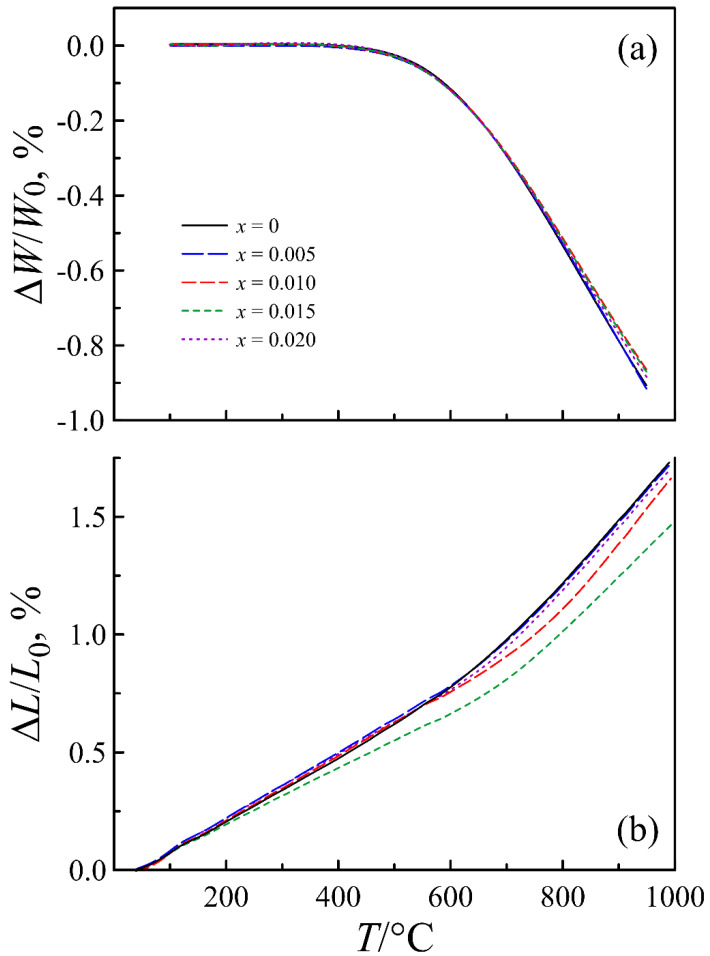
Relative weight change (**a**) and relative elongation (**b**) of a ceramic sample of La_0.5−*x*_Sr_0.5_FeO_3−*δ*_ as functions of temperature.

Figure 3 demonstrates that the nominal deficiency in the A-sublattice similarly affects the thermal properties of La_0.5−_*_x_*Sr_0.5_FeO_3−*δ*_ oxides. The results of both thermogravimetry and dilatometry show the extreme nature of the measured characteristics versus *x*. Thermogravimetric curves show an intensive decrease in the sample weight at heating above ~475 °C, which is associated with oxygen release from the crystalline lattice of the oxides and a respective decrease in the average oxidation state of iron ions. As can be seen in Figure 3a, the *x* = 0.1 composition exhibits the lowest weight variation with temperature, which is consistent with the above discussion.

In turn, the observed decrease in the oxygen homogeneity range, as can be expected, causes an improvement in the thermomechanical properties of ferrite, which is demonstrated by the results of the dilatometric measurements in Figure 3b. In particular, the results show that the relative elongation upon heating of a ceramic sample of the *x* = 0.01 composition is noticeably smaller than that of the *x* = 0 composition.

**Figure 4:**
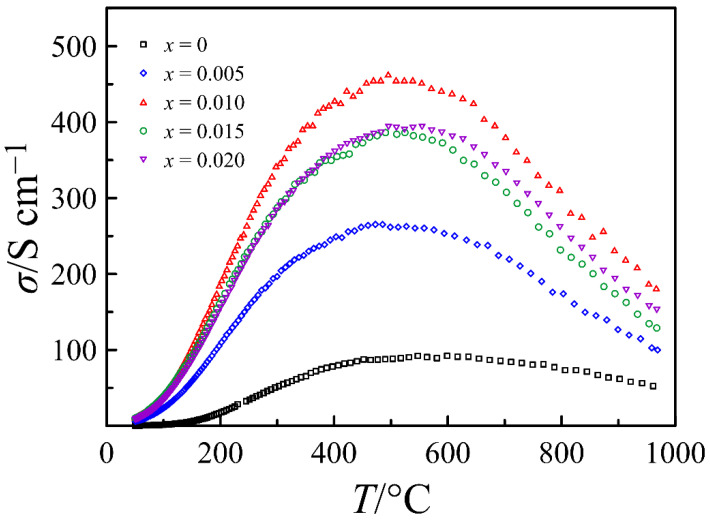
Electrical conductivity of La_0.5−*x*_Sr_0.5_FeO_3−*δ*_ ceramics sintered at 1300 °C, measured in air versus temperature.

Figure 4 presents the electrical conductivity of La_0.5−_*_x_*Sr_0.5_FeO_3−*δ*_ measured as a function of temperature in the air atmosphere. The hole conductivity is known to be dominant in perovskite-type ferrites under oxidizing conditions [31]. Therefore, an increase in the conductivity with temperature increase up to ~475 °C indicates a temperature-activated mechanism of the hole transport, while its decrease with further heating is a result of a decrease in p-type carrier concentration due to oxygen removal from the oxide, as illustrated by Figure 3a. The data in Figure 4 reveal a strong impact of the deficiency in the A-sublattice on the electrical conductivity of La_0.5−_*_x_*Sr_0.5_FeO_3−*δ*_. The maximum conductivity value obtained for the *x* = 0.01 composition is more than four times higher than that for the oxide with the complete A-sublattice. In order to clarify the origin of such extraordinal dependence, the morphology of the ceramic samples was studied by scanning electron microscopy.

**Figure 5:**
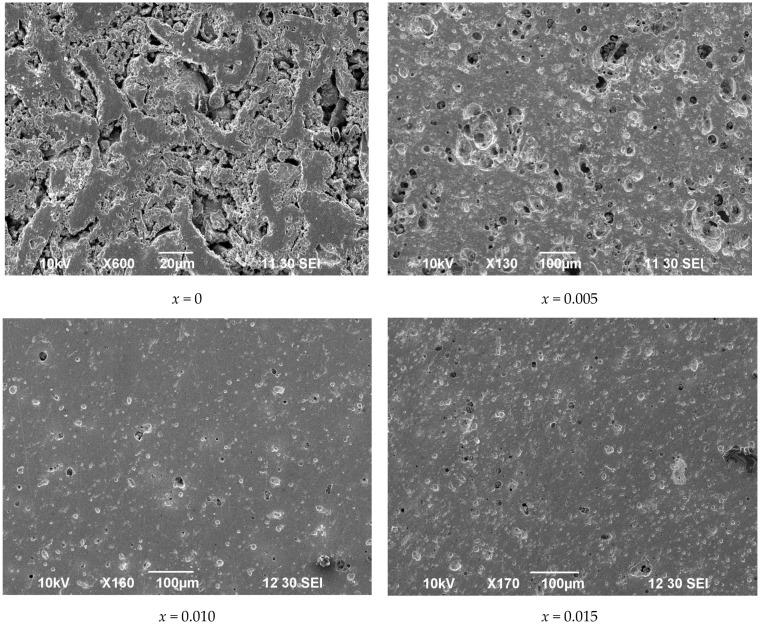
Scanning electron microscopy images of La_0.5−*x*_Sr_0.5_FeO_3−*δ*_ ceramics sintered at 1300 °C.

The SEM images in Figure 5 clearly show that, despite the identical conditions of the materials synthesis and the ceramic sintering, the morphology of the obtained samples differs significantly. It is obvious that the cation deficiency increase stimulates ceramic improvement. In particular, it leads to lower porosity and higher density, as can be seen in Table 2. It should be noted that similar effects have been reported earlier for other perovskite-type oxides [32,33]. Although the mechanism of the phenomenon needs further detailed study, it can be assumed that the vacancies in the A-sublattice facilitate the cation transport in the oxides and thus promote the formation of compact ceramics at lower temperatures.

**Figure 6:**
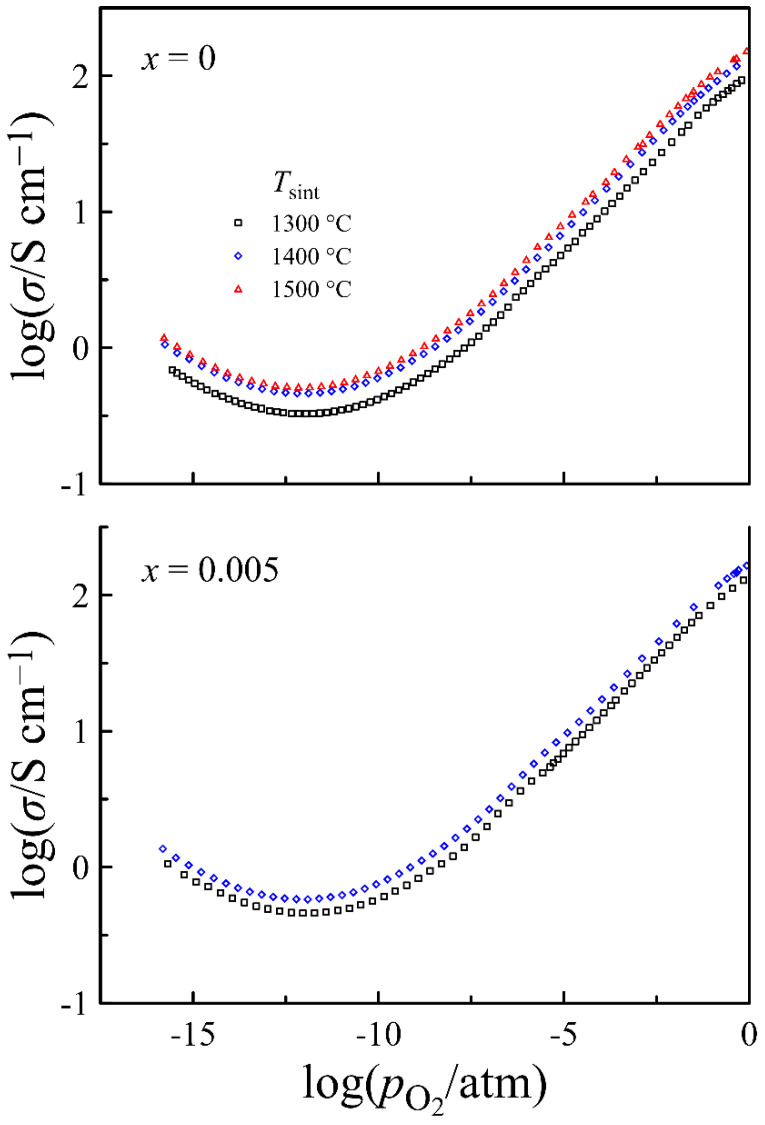
Electrical conductivity of La_0.5−*x*_Sr_0.5_FeO_3−*δ*_ ceramics sintered at different temperatures, measured at 950 °C versus oxygen partial pressure.

Since the better conductivity of the *x* = 0.1 composition compared to that of *x* = 0 and *x* = 0.005 is largely attributed to a higher density of the corresponding ceramics, an attempt was made to increase the ceramic density of the *x* = 0 and *x* = 0.005 compositions by sintering at higher temperatures and to trace the effect of the treatment on electrical conductivity. Figure 6 shows the electrical conductivity as a function of oxygen partial pressure at 950 °C, measured on the samples with the *x* = 0 and *x* = 0.005 compositions, sintered at 1300, 1400, and 1500 °C. It can be seen that an increase in sintering temperature leads to a uniform conductivity increase over the entire experimental range of $p_{O_{2}}$, which seems natural as a result of the material density improvement. A comparison of the data in Table 2 and Figure 6 shows that an increase in sintering temperature from 1300 to 1400 °C results in a noticeable increase in density of the *x* = 0 ceramics and in a ~30% increase in its conductivity, while an additional heat treatment at 1500 °C gives less increase in ceramic density and only ~10% increase in conductivity. Similarly, the greatest impact on both the ceramic density and the conductivity of the *x* = 0.005 composition is provided by an increase in sintering temperature from 1300 to 1400 °C. Since a further increase in sintering temperature to 1500 °C did not improve the ceramic density, its conductivity was not measured. As far as the density of the *x* = 0 and *x* = 0.005 samples sintered at 1500 and 1400 °C, respectively, was high enough and comparable with that of samples with higher *x* values sintered at 1300 °C, these samples were used for a detailed conductivity measurement.

**Figure 7:**
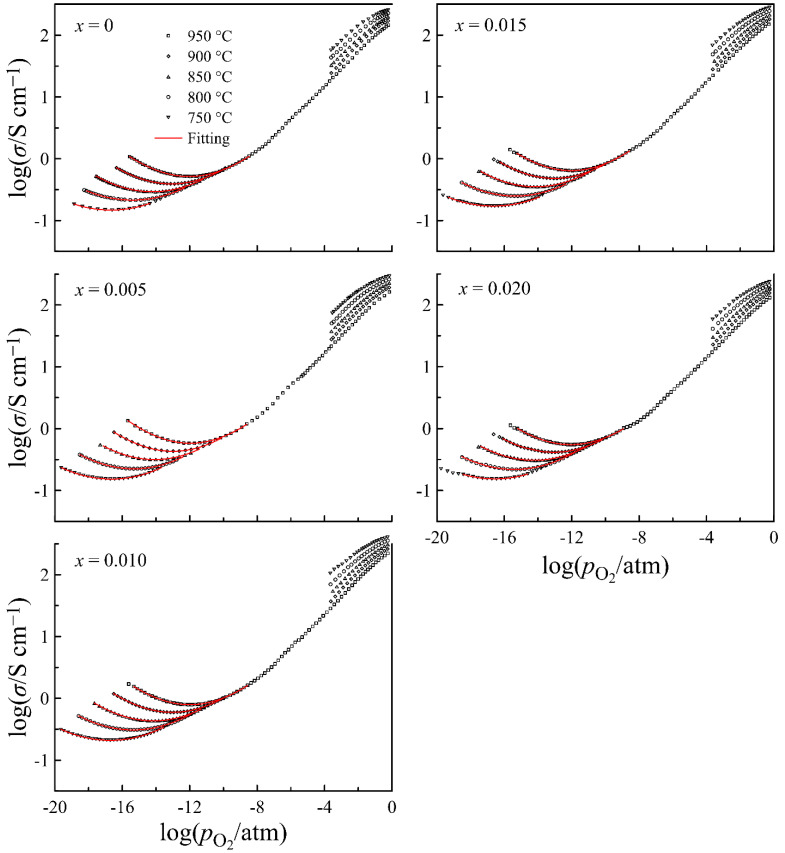
Electrical conductivity of dense La_0.5−*x*_Sr_0.5_FeO_3−*δ*_ ceramics (≥90%) as a function of oxygen partial pressure at different temperatures. Solid lines represent the results of Equation (1) approximation to the experimental data.

**Figure 8:**
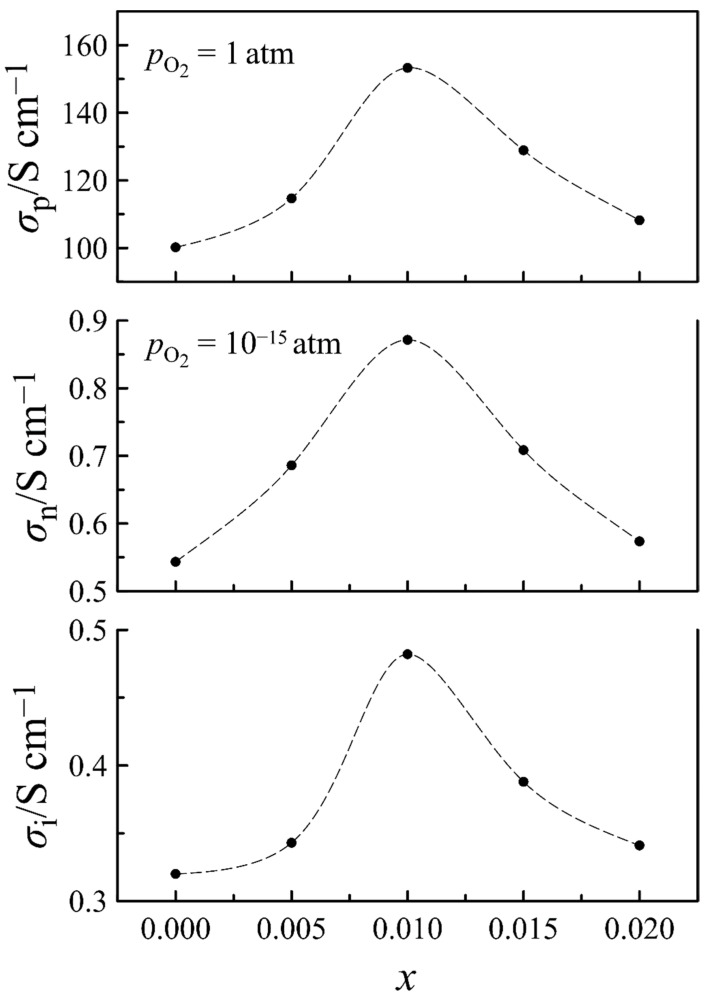
Partial contributions to the conductivity of La_0.5−*x*_Sr_0.5_FeO_3−*δ*_ as functions of cation deficiency. Dashed lines are guides for the eyes.

Figure 7 shows the electrical conductivity of La_0.5−_*_x_*Sr_0.5_FeO_3−*δ*_ versus oxygen partial pressure at different temperatures, obtained on the specimens with comparable density. The shape of conductivity isotherms is similar to that for the other (La,Sr)FeO_3−*δ*_ compounds [27,31]. A decrease in the conductivity upon a decrease in partial pressure of oxygen indicates the predominance of p-type carriers in the charge transfer, whereas its increase after the minimum with a further decrease in $p_{O_{2}}$ manifests the prevailing contribution of n-type carriers to the total conductivity. A smooth shape of the conductivity minima in isotherms implies the presence of ion conductivity. Thus, the shape of conductivity isotherms suggests the presence of contributions from the charge carriers of three types, having different dependence on oxygen partial pressure. Therefore, the total conductivity in the vicinity of the minima can be approximated by the well-known expression:

(1) $$\sigma \left(T,p_{O_{2}}\right)=\sigma_{i}\left(T\right)+\sigma_{n}^{0}\left(T\right)p_{O_{2}}^{-\frac{1}{4}}+\sigma_{p}^{0}\left(T\right)p_{O_{2}}^{+\frac{1}{4}}$$

where $\sigma_{i}(T)$ is the ion conductivity, whereas $\sigma_{n}^{0}(T)$ and $\sigma_{p}^{0}(T)$ are the n- and p-type conductivity, respectively, extrapolated to $p_{O_{2}}$= 1 atm. The results of Equation (1) approximation to the experimental data, shown in Figure 7 by solid lines, follow perfectly the experimental data, and errors in the determination of $\sigma_{i}$, $\sigma_{n}^{0}$, and $\sigma_{p}^{0}$ parameters do not exceed 2%. This characterizes the obtained parameters of the total conductivity as reliable. Their values can be found in Table S1 in the Electronic Supplementary Information (ESI). Using these data, partial contributions to conductivity from different charge carriers are shown in the form of Arrhenius plots in Figure S1 in ESI. Taking into account that the electron and hole conductivity depend on oxygen partial pressure, these contributions are shown at practically useful $p_{O_{2}}$ values of 10^−15^ and 1 atm, respectively. A negative slope of hole conductivity plots is associated with the exothermal character of the reaction of oxygen incorporation in La_0.5−_*_x_*Sr_0.5_FeO_3−*δ*_, resulting in the formation of p-type carriers. The linear approximation of the dependences shows that the activation energy of electron conductivity is 2.4 ± 0.1 eV, being unaffected by the cation deficiency, whereas the activation energy of ion conductivity varies between 0.67 ± 0.01 and 0.71 ± 0.01 eV. These values, as well as the magnitude of partial conductivities, are in good agreement with the respective literature data [11,27,34]. In order to trace the effect of the cation deficiency on partial conductivities, the respective data for 950 °C are depicted in Figure 8. It can be seen that all three curves demonstrate maximum at *x* = 0.01, similar to the unit cell volume in Figure 2. Taking into account that the observed decrease in conductivity at *x* > 0.01 is probably associated with the progressive formation of the high resistive impurity phase, the greatest interest is caused by the increase in the conductivity, with *x* increasing up to 0.01. For instance, the ion conductivity of the *x* = 0.01 composition is one and a half times higher than that of *x* = 0. In order to clarify the origin of the effect, a more detailed analysis of charge transport in the *x* = 0 and *x* = 0.01 compositions, based on the results of defect chemistry analyses in the oxides, have been performed.

### 3.2. Oxygen Content and Defect Equilibrium

**Figure 9:**
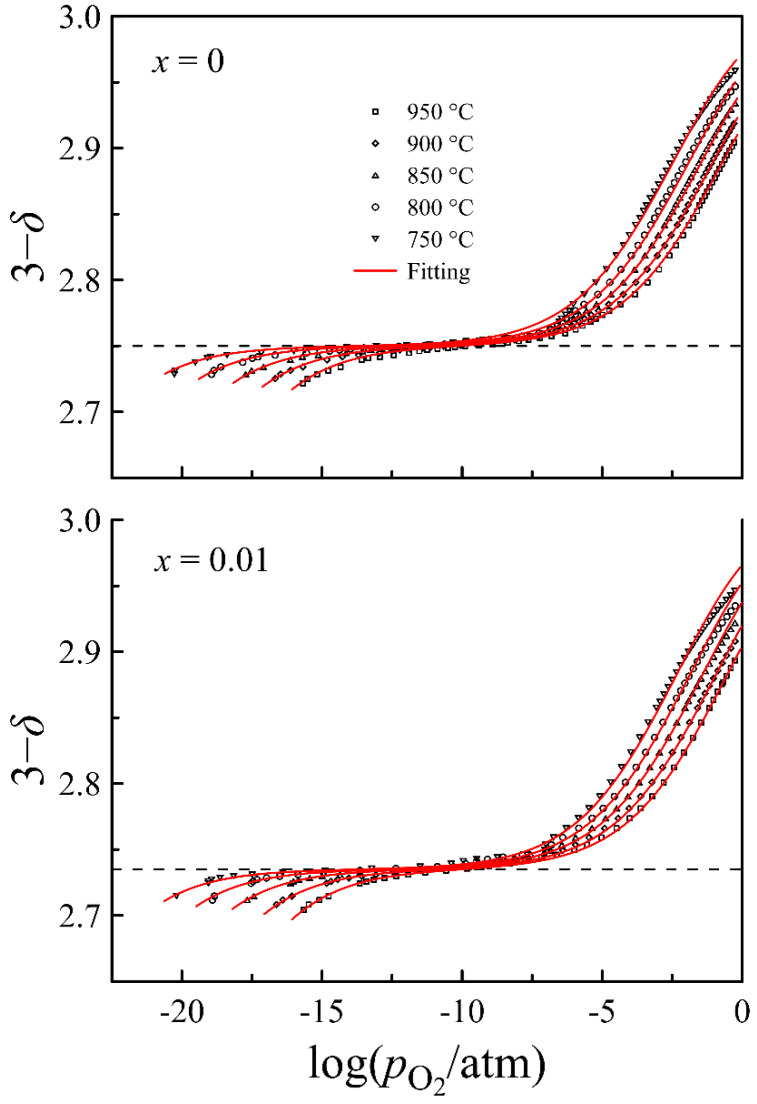
Oxygen content in La_0.5−*x*_Sr_0.5_FeO_3−*δ*_ (*x* = 0 and 0.01) as a function of oxygen partial pressure at different temperatures. Solid lines represent results of model calculation.

In Figure 9, experimental data on the oxygen content in La_0.5_Sr_0.5_FeO_3−*δ*_ and La_0.49_Sr_0.5_FeO_3−*δ*_ versus oxygen partial pressure at different temperatures are shown by symbols. Dashed lines represent the oxygen content, providing the average oxidation state of iron ions in the oxides 3+, according to the formulas: ${\mathrm{La}}_{0.5}^{3+}{\mathrm{Sr}}_{0.5}^{2+}{\mathrm{Fe}}_{}^{3+}O_{2.75}^{2-}$ and ${\mathrm{La}}_{0.49}^{3+}{\mathrm{Sr}}_{0.5}^{2+}{\mathrm{Fe}}_{}^{3+}O_{2.735}^{2-}$. An increase in the oxygen content from these levels results in the appearance of Fe^4+^ ions, which can be considered as Fe^3+^ ions with p-type carriers localized on them. A decrease in the oxygen content is accompanied by Fe^3+^ ions reduction to Fe^2+^, which can be considered as Fe^3+^ ions with n-type carriers localized on them [31]. Taking into account the oxidation states of the cations comprised in La_0.5−_*_x_*Sr_0.5_FeO_3−*δ*_, the generalized chemical formula of ferrites can be written as: ${\mathrm{La}}_{0.5-x}^{3+}{\mathrm{Sr}}_{0.5}^{2+}{\mathrm{Fe}}_{n}^{2+}{\mathrm{Fe}}_{a}^{3+}{\mathrm{Fe}}_{p}^{4+}O_{3-\delta }^{2-}$, where *n*, *a*, and *p* denote the concentrations of the corresponding iron ions. An increase in $p_{O_{2}}$ in the gas phase is accompanied by oxygen incorporation into the oxide. The oxidation reaction and the expression for its equilibrium constant can be written as:

$$2{\mathrm{Fe}}^{3+}+V_{O}+\frac{1}{2}O_{2}=2{\mathrm{Fe}}^{4+}+O^{2-}$$

(2) $$K_{\mathrm{ox}}=\frac{{\left[{\mathrm{Fe}}^{4+}\right]}^{2}\cdot \left[O^{2-}\right]}{{\left[{\mathrm{Fe}}^{3+}\right]}^{2}\cdot \left[V_{O}\right]}p_{O_{2}}^{-1/2}=\frac{p^{2}\cdot \left(3-\delta \right)}{a^{2}\cdot \delta }p_{O_{2}}^{-1/2}$$

Fe^3+^ ions are also involved in the charge disproportionation reaction:

$$2{\mathrm{Fe}}^{3+}={\mathrm{Fe}}^{4+}+{\mathrm{Fe}}^{2+}$$

(3) $$K_{d}=\frac{\left[{\mathrm{Fe}}^{2+}\right]\cdot \left[{\mathrm{Fe}}^{4+}\right]}{{\left[{\mathrm{Fe}}^{3+}\right]}^{2}}=\frac{n\cdot p}{a^{2}}$$

Additionally, the requirements to preserve the iron sublattice site and electroneutrality should be taken into account:

*a* + *n* + *p* = 1, (4)

*n* = 2*δ* − 0.5 − 3*x* + *p* (5)

The solution of the system of Equations (3)–(5) gives the relationship between the concentrations of the iron ions in different oxidation states *a*, *n*, and *p* and the oxygen content (3 − *δ*). The relationship between $p_{O_{2}}$ and the oxygen content in the oxide can be obtained from Equation (2):

(6) $$p_{O_{2}}^{1/2}=\frac{p^{2}\cdot \left(3-\delta \right)}{a^{2}\cdot \delta \cdot K_{\mathrm{ox}}}$$

**Figure 10:**
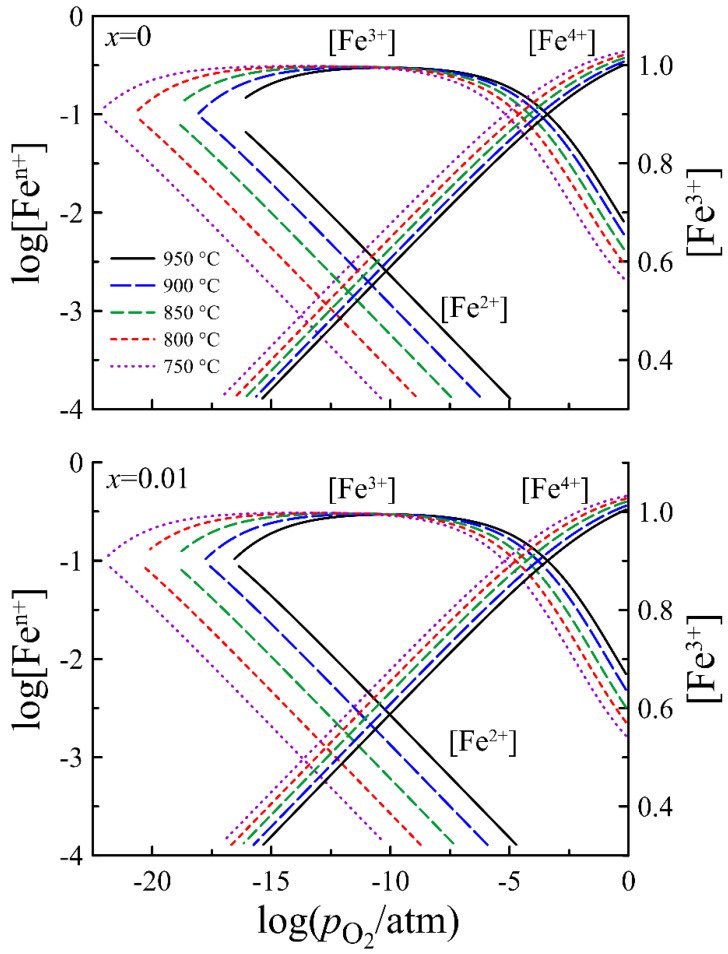
Concentration of iron ions in different oxidation states in La_0.5−*x*_Sr_0.5_FeO_3−*δ*_ (*x* = 0 and 0.01) as a function of oxygen partial pressure at different temperatures.

The combination of the above equations results in a rather long expression which was used for the approximation to the oxygen content data. The calculated plots of (3 − *δ*) versus $p_{O_{2}}$ presented in Figure 9 by solid lines agree well with the experimental data, which indicates the correct determination of the equilibrium constants for the defect formation reactions in (2) and (3). The relation between the reaction equilibrium constants and the corresponding thermodynamic parameters is established by the well-known expression:

(7) $$K_{j}=\exp\left(-\frac{\Delta G_{j}^{0}}{RT}\right)=\exp\left(-\frac{\Delta H_{j}^{0}}{RT}+\frac{\Delta S_{j}^{0}}{R}\right)$$

where R is the gas constant, and $\Delta G_{j}^{0}$, $\Delta H_{j}^{0}$, and $\Delta S_{j}^{0}$ are the standard free Gibbs energy, enthalpy, and entropy changes for the defect formation reactions, respectively. Table 3 contains $\Delta H_{}^{0}$ and $\Delta S_{}^{0}$ values for the oxidation and charge disproportionation reactions, which, according to Equation (7), were determined from the slopes of van’t Hoff’s plots in Figure S2 (see ESI) for the respective equilibrium constants [31]. The calculated concentrations of the iron ions in different oxidation states are shown in Figure 10. The data for *x* = 0 and *x* = 0.01 look very similar because the difference in compositions is rather small.

### 3.3. Mobility of Charge Carriers

The obtained data on the concentration of the charge carriers allow us to analyze the electrical conductivity of La_0.5−_*_x_*Sr_0.5_FeO_3−*δ*_ in the entire experimental range, taking into account that it comprises the contributions from charge carriers of three types:

(8) $$\sigma =\sigma_{i}+\sigma_{n}+\sigma_{p}$$

It is assumed that all oxygen ions (3 − *δ*) contribute to ion conductivity by migration over the anion vacancies. Thus, the expression for ion conductivity can be written as follows:

(9) $$\sigma_{i}=N\cdot e\cdot 2\cdot \left(3-\delta \right)\cdot \mu_{i}$$

where *N* is the number of La_0.5−_*_x_*Sr_0.5_FeO_3−*δ*_ unit cells per 1 cm^3^, *e* is the unit charge, multiplier 2 takes into account the charge of the oxygen ion, and $\mu_{i}$ is the mobility of the oxygen ions:

(10) $$\mu_{i}=\mu_{i}^{0}\cdot \delta$$

where $\mu_{i}^{0}$ is the parameter of the oxygen ion mobility unaffected by the oxygen content in the oxide.

The electron and hole conductivity in perovskite-type ferrites is known to occur by the polaron hopping mechanism [31]. Therefore, partial contributions to electrical conductivity from n- and p-type carriers can be expressed as follows:

(11) $$\sigma_{n}=N\cdot e\cdot \left[{\mathrm{Fe}}^{2+}\right]\cdot \mu_{n}$$

(12) $$\sigma_{p}=N\cdot e\cdot \left[{\mathrm{Fe}}^{4+}\right]\cdot \mu_{p}$$

where $\left[{\mathrm{Fe}}^{2+}\right]$ and $\left[{\mathrm{Fe}}^{4+}\right]$ designate the concentrations, whereas $\mu_{n}$ and $\mu_{p}$ denote the mobility of n- and p-type carriers, respectively. Taking into account that the migration of the electronic carriers occurs via Fe^3+^ ions, the expression for the mobility of n- and p-type carriers can be written as follows:

(13) $$\mu_{n}=\mu_{n}^{0}\cdot \left[{\mathrm{Fe}}^{3+}\right]$$

(14) $$\mu_{p}=\mu_{p}^{0}\cdot \left[{\mathrm{Fe}}^{3+}\right]$$

where $\mu_{n}^{0}$ and $\mu_{p}^{0}$ are the parameters of electron and hole mobility, which are expected to be unaffected by the oxygen content. Equation (13) implies a small decrease in the mobility of n-type carriers as $p_{O_{2}}$ decreases under reducing conditions due to a weak decrease in the concentration of Fe^3+^ ions with oxygen removal from La_0.5−_*_x_*Sr_0.5_FeO_3−*δ*_, which can be observed in Figure 10. Similarly, the behavior of the concentration of Fe^3+^ ions in Figure 10 should result in a decrease in the mobility of p-type carriers with increasing oxygen partial pressure under oxidizing conditions. In contrast, a number of studies have reported a significant increase in the hole mobility upon an increase in the oxygen content in perovskite-type ferrites [35,36,37,38,39,40]. It is assumed that the effect is partially attributed to an increase in the integrity of the Fe–O–Fe hole transport network with the filling of oxygen vacancies. There is also an assumption that oxygen vacancies make several nearby Fe^3+^ ions inaccessible for the hole jump, so an increase in the oxygen content involves more iron ions in the hole transport and enhances mobility [35]. Usually, the mobility of p-type carriers can be approximated well by linear dependence on the concentration of carriers (or oxygen content) [41,42]. In some cases, however, the best description needs the use of a second degree polynomial [43,44]. The latter approach gave better results in preliminary tests; therefore, the following expression was used for the hole mobility approximation to the experimental data:

(15) $$\mu_{p}=\left[{\mathrm{Fe}}^{3+}\right]\cdot \left(\mu_{p}^{0}+\mu_{p}^{1}\cdot \left[{\mathrm{Fe}}^{4+}\right]+\mu_{p}^{2}\cdot {\left[{\mathrm{Fe}}^{4+}\right]}^{2}\right)$$

where $\mu_{p}^{0}$, $\mu_{p}^{1}$, and $\mu_{p}^{2}$ are constants. Thus, Equation (8) for total conductivity acquires the following form:

(16) $$\sigma =N\cdot e\left(2\cdot \delta \cdot \left(3-\delta \right)\cdot \mu_{i}^{0}+\left[{\mathrm{Fe}}^{2+}\right]\cdot \left[{\mathrm{Fe}}^{3+}\right]\cdot \mu_{n}^{0}+\left[{\mathrm{Fe}}^{4+}\right]\cdot \left[{\mathrm{Fe}}^{3+}\right]\cdot \left(\mu_{p}^{0}+\mu_{p}^{1}\cdot \left[{\mathrm{Fe}}^{4+}\right]+\mu_{p}^{2}\cdot {\left[{\mathrm{Fe}}^{4+}\right]}^{2}\right)\right)$$

**Figure 11:**
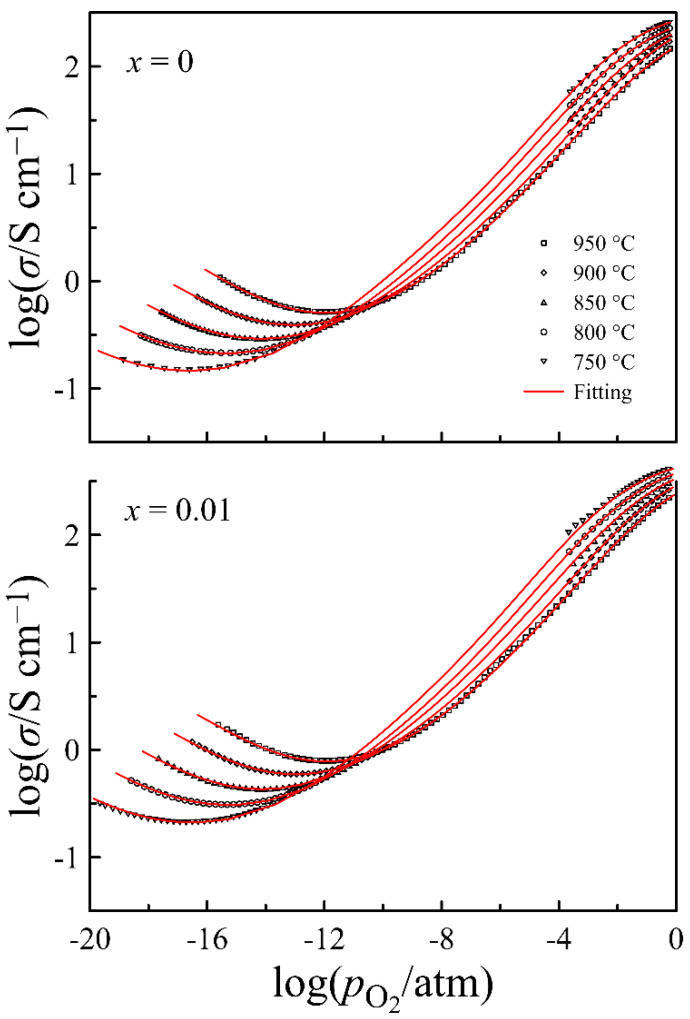
Electrical conductivity of La_0.5−*x*_Sr_0.5_FeO_3−*δ*_ (*x* = 0 and 0.01) as a function of oxygen partial pressure at different temperatures. Solid lines represent the results of Eq.

**Figure 12:**
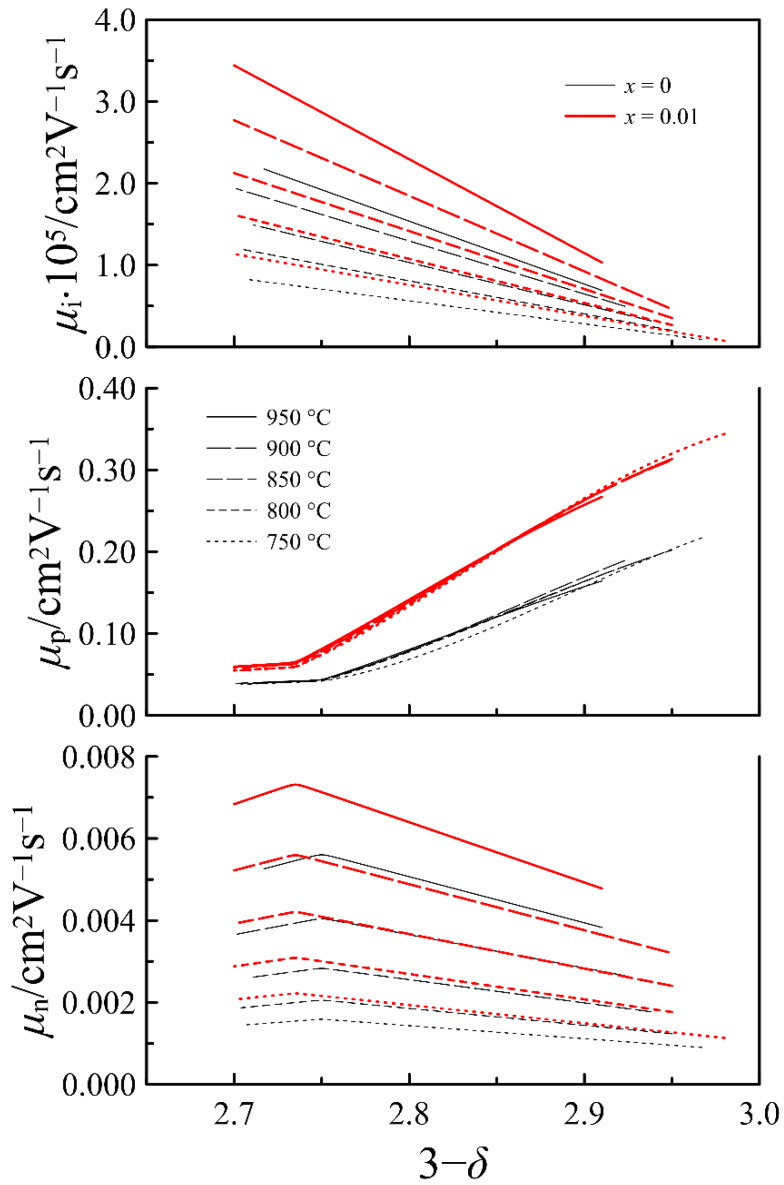
Mobility of oxygen ions, holes, and electrons in La_0.5−*x*_Sr_0.5_FeO_3−*δ*_ (*x* = 0 and 0.01) as a function of oxygen content in oxides at different temperatures.

Solid lines in Figure 11, which present the results of calculations with Equation (16), follow the experimental data fairly well. The obtained mobility parameters for oxygen ions, electrons, and holes are collected in Table S2 of the ESI. In Figure 12, the mobility of the charge carriers in the *x* = 0 and *x* = 0.01 compositions is shown as a function of the oxygen content in La_0.5−_*_x_*Sr_0.5_FeO_3−*δ*_. It can be seen that an increase in the cation deficiency enhances the mobility of all the charge carriers. The increase in the mobility is ~25% for n-type carriers, ~50% for oxygen ions, and ~70% for p-type carriers. An increase in the oxygen ion mobility can be understood as a result of an expansion of some bottlenecks in the pathway of the oxygen ions which goes between the two A-cations and the iron ion. The contribution of the cation deficiency to enhancing the mobility of n- and p-type carriers can be associated with its effect on the local symmetry of the crystalline lattice. Taking into account the favorable effect of the cation deficiency on the lattice symmetry noted in Table 1, we can assume that the formation of the cation vacancies can partially compensate for local distortions provided by the presence of the oxygen vacancies and is unfavorable for the mobility of electronic carriers. The Arrhenius plots in Figure S3 were used to determine the activation energy for the mobility of the charge carriers. In general, the obtained data on the charge carrier mobility in Figure 12 and the values of their activation energy presented in Table 4 are in a reasonable agreement with the literature data [35,43,45].

### 3.4. Evidence of SrFe_12_O_19_ Impurity Formation

**Figure 13:**
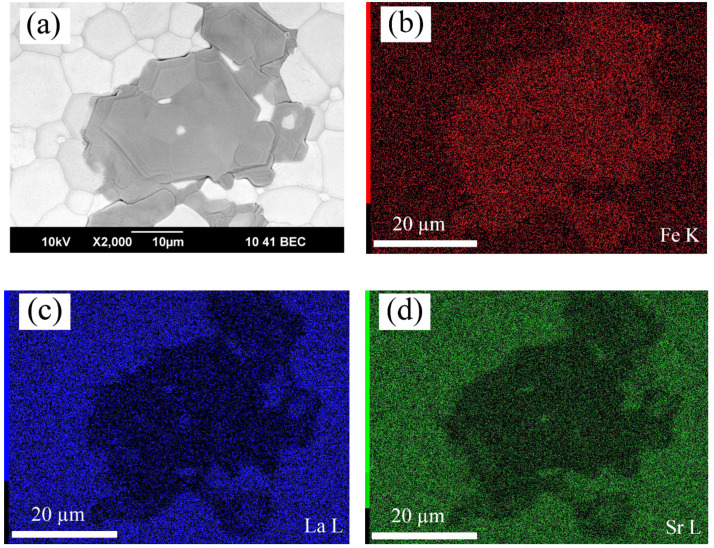
SEM (**a**) and EDX(**b**–**d**) images of La_0.5−*x*_Sr_0.5_FeO_3−*δ*_ (*x* = 0.02) ceramics subjected to heat treatment at 1300 °C for 2 h.

**Figure 14:**
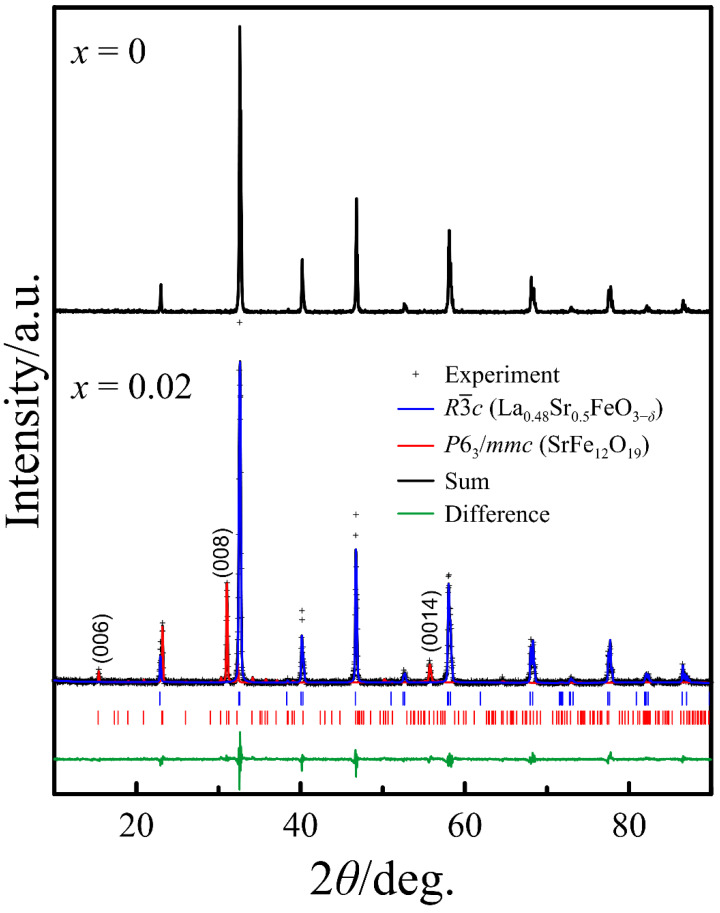
XRD patterns of the surface of ceramic discs with x = 0 and 0.02 compositions. Preferred orientation is along c-axis.

The SEM analysis of the ceramic samples was planned with the aim of evaluating the impact of the nominal cation deficiency on the grain size of the ceramics. The examination of the SEM images obtained on the polished ceramic specimens subjected to thermal treatment at 1300 °C detected a considerable difference between the *x* = 0 and *x* = 0.02 compositions. In contrast to the homogeneous surface of the former sample, the surface of the latter contained contrasting spots, as can be seen in Figure 13a. The EDX analysis of the spots revealed a lower strontium and lanthanum content and an excessive iron content, as demonstrated in Figure 13b–d. Such a variation in the composition can imply that contrasting areas on the ceramics consist of (Sr,La)Fe_12_O_19_ hexaferrite. The possibility of such an impurity formation in the perovskite-type ferrites with a nominal cation deficiency in the A-sublattice has been reported in [22]. This work indicates the difficulty of hexaferrite detection by the XRD technique, and the necessity to use high-energy synchrotron radiation for this purpose. Another paper notes that the presence of strontium hexaferrite in perovskite-type ferrites in quantities below 2 wt.% is practically undetectable by X-ray diffraction [46]. Nevertheless, as an impurity, presumably consisting of (Sr,La)Fe_12_O_19_, appeared on the ceramic surface in a considerable amount, an attempt to determine its structure by XRD analysis was made. Figure 14 shows for comparison the XRD patterns obtained from the surface of the ceramic samples with *x* = 0 and *x* = 0.02 compositions subjected to identical thermal treatment. It can be seen that the pattern of the latter sample contains a number of pretty strong reflexes of an additional phase. At first glance, the set of additional reflexes seems to be rather scarce for (Sr,La)Fe_12_O_19._ Nevertheless, taking into account that the thickness of the segregations is very small, one can assume the preferred orientation of the respective crystals. The observation of only the peaks (006), (008), and (0014) in the powder X-ray diffraction pattern indicates the texture of the phase and its predominant growth when the *c*-axis of the lattice is located perpendicular to the sample surface. Indeed, the X-ray diffraction patterns obtained from thin films of SrFe_12_O_19_ with the *c*-axis orientation were shown to have a very similar set of reflexes [47,48]. Rietveld refinement of the XRD pattern confirms the presence of (Sr,La)Fe_12_O_19_ crystals with a preferred orientation along the *c*-axis on the surface of ceramics with the *x* = 0.02 composition, as can be seen in Figure 14. This, in combination with all the experimental data above, supports our initial assumption that the maximal cation deficiency in the A-sublattice of La_0.5−_*_x_*Sr_0.5_FeO_3−*δ*_ cannot exceed 0.01. A little in excess of this limit results in the appearance of a small amount of (Sr,La)Fe_12_O_19_ impurity, which is probably located on the grain boundaries of the perovskite phase and cannot be detected by XRD. Heat treatment of the ceramics at temperatures above 1206 °C can result in the formation of a liquid phase in the impurity [49]. This facilitates the impurity migration and its concentration on the ceramic surface in an amount sufficient for detection by the XRD method. Moreover, the presence of a liquid phase should promote densification of the ceramics, which is consistent with the SEM analysis data in Figure 5 and Table 2. Although the *x* = 0.01 composition demonstrates the best transport characteristics, it cannot be excluded that the cation deficiency limit is slightly below 0.01, and the x = 0.01 ceramics sintered at 1300 °C achieved high density with the help of liquid phase traces.

## 4. Conclusions

The impact of the deficiency in the A-sublattice of La_0.5−_*_x_*Sr_0.5_FeO_3−*δ*_ oxides on the structure, thermomechanical, and transport properties was studied in the light of their potential application as materials of SOFC cathodes and oxygen membranes. An increase in the nominal deficiency from 0 to 0.02 was found to result in a nonlinear effect on the properties of La_0.5−_*_x_*Sr_0.5_FeO_3−*δ*_. An increase in the *x* value from 0 to 0.01 induces the formation of the cation vacancies in the A-sublattice, which is reflected in an increase in the unit cell volume and in electrical conductivity. An attempt to increase the cation deficiency above *x* = 0.01 was found to give the opposite effect due to (La,Sr)Fe_12_O_19_ impurity phase formation, which leads to a decrease in the number of vacancies in the A-sublattice. Therefore, the *x* = 0 and *x* = 0.01 compositions of La_0.5−_*_x_*Sr_0.5_FeO_3−*δ*_ were considered to be the most interesting and subjected to a more profound study. The electrical conductivity and the oxygen content were measured in these compositions as a function of the oxygen partial pressure, varying from 10^−19^ to 0.5 atm at 750–950 °C. The obtained data were employed for the combined analysis of the defect equilibrium and charge transfer. According to the results of this analysis, the revealed increase in the conductivity in response to the increase in the cation deficiency from 0 to 0.01 was conditioned by an increase in the mobility of the charge carriers. The introduction of a 1% deficiency in the A-sublattice of La_0.5−_*_x_*Sr_0.5_FeO_3−*δ*_ can be recommended as an effective means to enhance both the oxygen-ion and the electron conductivity.

## Figures and Tables

**Table 1:** Structural parameters of La_0.5−_*_x_*Sr_0.5_FeO_3−*δ*_ refined in Rhombohedral setting of $R\bar{3}C$ S.G.

*x*	*a*, Å	*α*, deg.	*V*, Å^3^	GOF	wR,%	wR_min_,%
0	5.487(6)	60.27	117.58	1.15	18.04	15.76
0.005	5.491(1)	60.25	117.75	1.17	19.98	16.25
0.010	5.495(2)	60.22	117.92	1.2	19.68	16.49
0.015	5.493(4)	60.23	117.84	1.16	18.65	16.16
0.020	5.484(0)	60.25	117.67	1.13	18.56	16.42

**Table 2:** Relative density of La_0.5−_*_x_*Sr_0.5_FeO_3−*δ*_ ceramics.

*x*	Sintering Temperature, °C	Density, %
0	1300	65.1
0	1400	87.5
**0** *	**1500**	**93.1**
0.005	1300	75.7
**0.005**	**1400**	**92.9**
0.005	1500	92.7
**0.01**	**1300**	**93.4**
**0.015**	**1300**	**92.9**
**0.020**	**1300**	**94.1**

* Information on ceramics used in conductivity measurements are highlighted by bold font.

**Table 3:** Thermodynamic parameters of the defect formation reactions in La_0.5−_*_x_*Sr_0.5_FeO_3−*δ.*_

x	$\Delta H_{\mathrm{ox}}^{0}/\mathrm{kJ}\cdot {\mathrm{mol}}^{-1}$	$\Delta S_{\mathrm{ox}}^{0}/J\cdot {\mathrm{mol}}^{-1}K^{-1}$	$\Delta H_{d}^{0}/\mathrm{kJ}\cdot {\mathrm{mol}}^{-1}$	$\Delta S_{d}^{0}/J\cdot {\mathrm{mol}}^{-1}K^{-1}$
0	−107 ± 2	−70 ± 2	111 ± 2	8 ± 2
0.01	−111 ± 1	−74 ± 1	114 ± 1	4.3 ± 0.4

**Table 4:** The migration energy values for oxygen ions, electrons, and holes in La_0.5−_*_x_*Sr_0.5_FeO_3−*δ.*_

*x*	*E*_mi_/eV	*E*_mn_/eV	*E*_mp_/eV
0	0.63 ± 0.03	0.78 ± 0.04	0.17 ± 0.02
0.01	0.69 ± 0.01	0.74 ± 0.01	0.126 ± 0.002

## Data Availability

All data included in this study are available upon request by contact with the corresponding author.

## Supplementary Materials

The following are available online at www.mdpi.com/article/10.3390/ma14205990/s1, Figure S1: Arrhenius plots for partial contributions to the conductivity of La0.5−xSr0.5FeO3−δ, obtained by the approximation of an Equation (1) to experimental data, Table S1: Parameters of electrical conductivity in La0.5−xSr0.5FeO3−δ, obtained by approximation of Eq. (1) to experimental data in vicinity of minima, Figure S2: Van’t Hoff’s plots of Kox and Kd. The solid lines represent linear approximations, Table S2: Model parameters, obtained by approximation to electrical conductivity of La_0.5−_*_x_*Sr_0.5_FeO_3−*δ*_. Figure S3: Arrhenius plots for mobility of n- and p-type carriers and that of oxygen ions in La_0.5−_*_x_*Sr_0.5_FeO_3−*δ*_.
