# Supplementary material for: Impact of A-Site Cation Deficiency on Charge Transport in La0.5−xSr0.5FeO3−δ
Source: Materials (Basel). 2021 Oct 12;14(20):5990. doi: 10.3390/ma14205990 (PMC8537157; doi:10.3390/ma14205990)
Supplement: Supplementary file 1 [file materials-14-05990-s001.zip › materials-1413009-supplementary.pdf]

materials

Impact of A-Site Cation Deficiency on Charge Transport in $La_{0.5-x}Sr_{0.5}FeO_{3-\delta}$

Oleg V. Merkulov [1,2,*], Ruslan R. Samigullin [3], Alexey A. Markov [1] and Mikhail V. Patrakeev [1]

Supplementary Materials

Table S1. Parameters of electrical conductivity in $La_{0.5-x}Sr_{0.5}FeO_{3-\delta}$, obtained byapproximation of Equation (1) to experimental data in vicinity of minima.

x	*T*/°C	σ_i^0/S cm-1	σ_n^0/S cm-1	σ_p^0/S cm-1
0*	950	0.320±0.001	(9.66±0.03)·10−5	100.2±0.4
	900	0.253±0.001	(3.73±0.01)·10−5	128.6±0.6
	850	0.201±0.001	(1.295±0.005)·10−5	162.9±0.8
	800	0.154±0.001	(4.22±0.04)·10−6	213±2
	750	0.108±0.001	(1.35±0.02)·10−6	293±3
0.005*	950	0.343±0.002	(1.22±0.01)·10−4	114.7±0.7
	900	0.272±0.001	(4.48±0.03)·10−5	141.1±0.9
	850	0.210±0.001	(1.60±0.01)·10−5	176±1
	800	0.154±0.001	(5.21±0.03)·10−6	237±2
	750	0.1088±0.0009	(1.52±0.02)·10−6	319±5
0.010*	950	0.482±0.003	(1.55±0.01)·10−4	153.3±0.9
	900	0.379±0.001	(5.95±0.02)·10−5	193.3±0.6
	850	0.289±0.001	(2.08±0.01)·10−5	247±1
	800	0.216±0.001	(6.72±0.04)·10−6	320±2
	750	0.154±0.001	(1.90±0.02)·10−6	425±4
0.015*	950	0.388±0.001	(1.26±0.01)·10−4	128.9±0.5
	900	0.305±0.001	(4.85±0.02)·10−5	159.1±0.9
	850	0.234±0.001	(1.70±0.01)·10−5	200±1
	800	0.176±0.001	(5.38±0.04)·10−6	253±2
	750	0.126±0.001	(1.62±0.02)·10−6	334±4
0.020*	950	0.341±0.002	(1.02±0.01)·10−4	108.2±0.9
	900	0.268±0.001	(3.87±0.02)·10−5	135±1
	850	0.209±0.002	(1.35±0.02)·10−5	176±2
	800	0.150±0.001	(5.14±0.04)·10−6	228±1
	750	0.110±0.001	(1.64±0.01)·10−6	303±2

* Data were obtained on the ceramic samples sintered at 1500 °C for *x* = 0, 1400 °C for *x* = 0.005, and 1300 °C for the rest compositions.

Table S2. Model parameters, obtained by approximation to electrical conductivity of $La_{0.5-x}Sr_{0.5}FeO_{3-\delta}$.

x	T, °C	$\mu_i^0 \cdot 10^5$	$\mu_n^0 \cdot 10^3$	$\mu_p^0 \cdot 10^2$	μ_p^1	μ_p^2
0	950	7.7±0.2	5.6±0.1	4.33±0.06	9.1±0.5	16±1
	900	6.48±0.07	4.06±0.05	4.35±0.05	7.5±0.3	24.8±0.7
	850	5.16±0.05	2.84±0.03	4.32±0.06	8.1±0.3	22.2±0.6
	800	4.04±0.07	2.06±0.07	4.2±0.1	8.2±0.6	23±1
	750	2.82±0.05	1.59±0.07	4.2±0.1	5.1±0.4	31.6±0.8
0.01	950	11.5±0.1	7.35±0.09	6.41±0.06	9.7±0.3	16.5±0.8
	900	9.24±0.06	5.62±0.05	6.36±0.05	9.0±0.2	20.2±0.4
	850	7.08±0.04	4.22±0.03	6.26±0.05	9.1±0.2	21.0±0.4
	800	5.38±0.04	3.10±0.04	5.91±0.06	9.8±0.2	22.2±0.5
	750	3.79±0.03	2.23±0.04	5.89±0.07	9.1±0.4	25±1

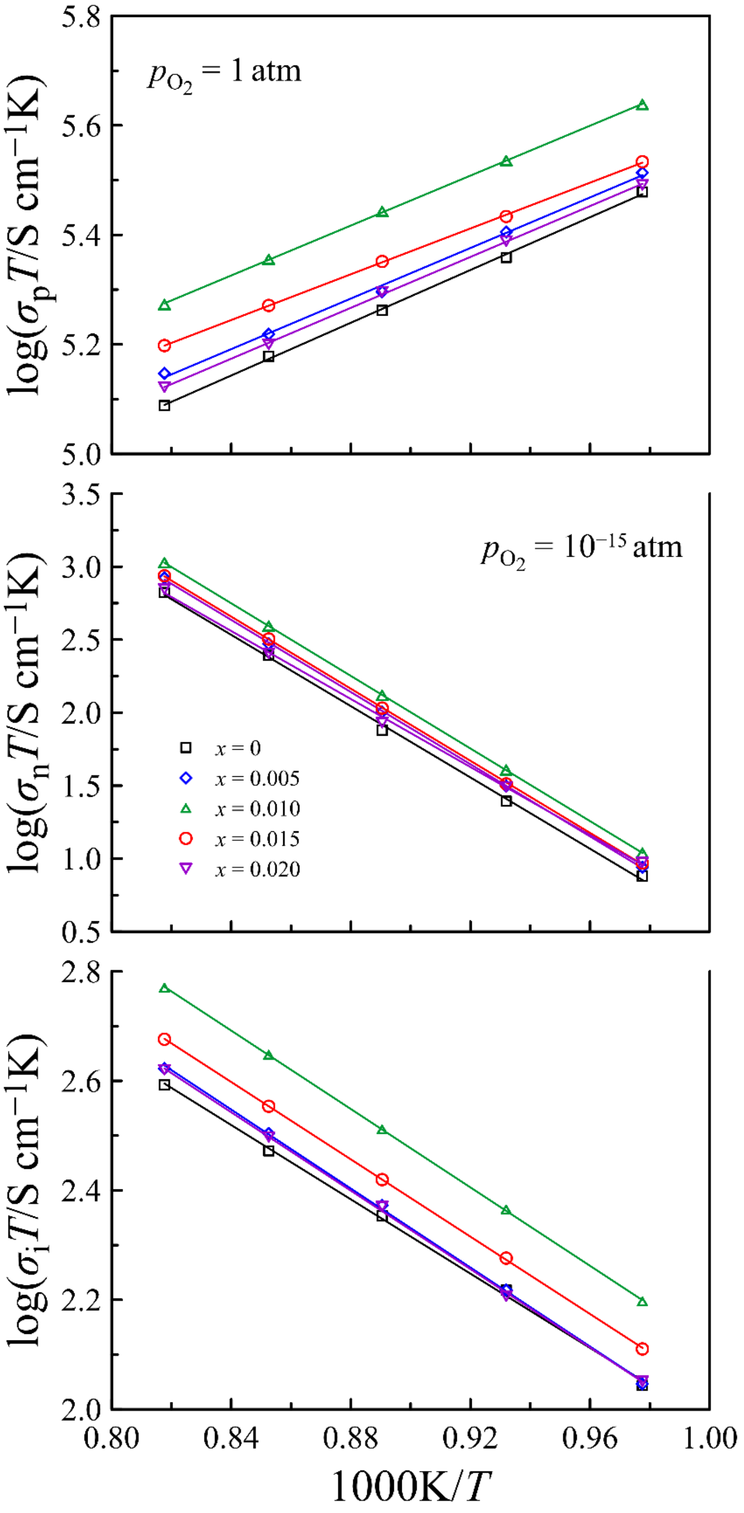

Figure S1. Arrhenius plots for partial contributions to the conductivity of $La_{0.5-x}Sr_{0.5}FeO_{3-\delta}$, obtained by the approximation of an Equation (1) to experimental data.

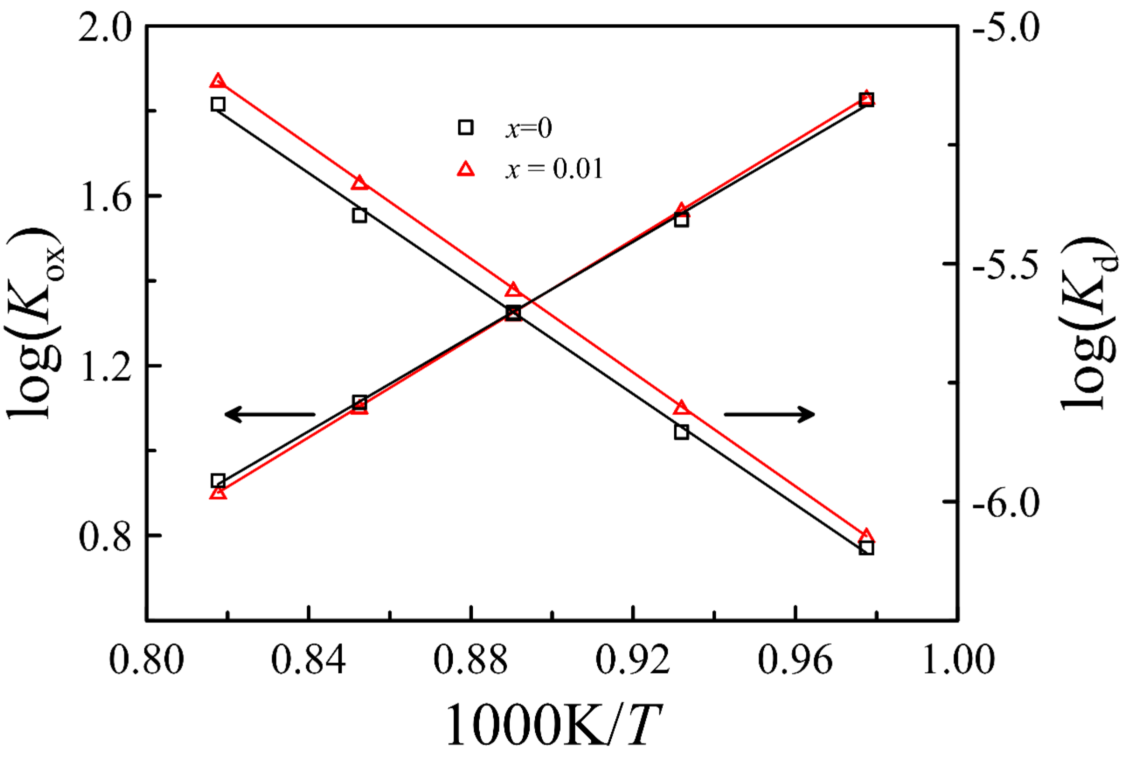

Figure S2. Van't Hoff's plots of K_{ox} and K_d. The solid lines represent linear approximations.
